# Why We Need to Take a Closer Look at Genetic Contributions to CYP3A Activity

**DOI:** 10.3389/fphar.2022.912618

**Published:** 2022-06-16

**Authors:** Qinglian Zhai, Maaike van der Lee, Teun van Gelder, Jesse J. Swen

**Affiliations:** Department of Clinical Pharmacy and Toxicology, Leiden University Medical Center, Leiden, Netherlands

**Keywords:** CYP3A locus, CYP3A4, CYP3A5, genetic variants, enzyme activity, missing heritability, pharmacogenomics

## Abstract

Cytochrome P450 3A (CYP3A) subfamily enzymes are involved in the metabolism of 40% of drugs in clinical use. Twin studies have indicated that 66% of the variability in CYP3A4 activity is hereditary. Yet, the complexity of the *CYP3A* locus and the lack of distinct drug metabolizer phenotypes has limited the identification and clinical application of CYP3A genetic variants compared to other Cytochrome P450 enzymes. In recent years evidence has emerged indicating that a substantial part of the missing heritability is caused by low frequency genetic variation. In this review, we outline the current pharmacogenomics knowledge of CYP3A activity and discuss potential future directions to improve our genetic knowledge and ability to explain CYP3A variability.

## Introduction

The Cytochrome P450 CYP3A subgroup forms the largest proportion of CYP protein in the human liver and small intestine (Shimada et al., 1994; Rendic, 2002) and is involved in the metabolism of up to 60% of currently used drugs (Plant, 2007; Zanger et al., 2008). The *CYP3A* locus is located on Chromosome seven and consists of four distinct genes *CYP3A4*, *CYP3A5*, *CYP3A7*, *CYP3A43*, and three pseudogenes (Finta and Zaphiropoulos, 2000; Gellner et al., 2001). Notably, the four coding genes share more than 85% of their amino-acid sequence and have a partially overlapping substrate spectrum (Williams et al., 2002). However, the contribution to drug metabolism varies considerably between the *CYP3A* genes (Ozdemir et al., 2000; Lamba et al., 2002). Of the entire CYP3A enzyme family, the CYP3A4 and CYP3A5 enzymes are the most abundantly expressed proteins accounting for more than 95% of total *CYP3A* mRNA pool (Koch et al., 2002). CYP3A7, on the other hand, is most prominent in fetal liver cells and slowly diminishes after birth. CYP3A43 expression is almost negligible compared to the other enzymes (<5% of total mRNA).

CYP3A activity is characterized by substantial inter-individual heterogeneity. For instance, CYP3A4, the predominant isoform of CYP3A, shows a 10–100-fold inter-individual variability in enzyme activity (Perera, 2010). One of the causes of the variability in CYP3A activity is the presence of genetic variants in the genes encoding the CYP3A enzymes. The clinical importance of several of these variants has indeed been reported in many patients (Evans and Relling, 1999; Zanger et al., 2008; Zanger and Schwab, 2013). Interestingly, there are currently only two haplotypes that are used in clinical practice, the CYP3A5*3 allele resulting in a complete loss-of-function, and the CYP3A4*22 allele resulting in a decreased enzyme activity (Kuehl et al., 2001; Wang et al., 2011). By combining CYP3A5*3 and CYP3A4*22 more than 60% and 20% of the observed variability in tacrolimus and cyclosporine trough blood level could be explained respectively (Elens et al., 2011). Recommendations on how to apply *CYP3A5* genotyping results to optimize drug and dose selection are included in clinical guidelines from the CPIC (Clinical Pharmacogenetic Implementation Consortium) (Birdwell et al., 2015) and for *CYP3A4* and *CYP3A5* by the DPWG (Dutch Pharmacogenomics Working Group) (Swen et al., 2011) (https://www.knmp.nl/index.php/media/1058).

Besides the genetic variants that form the CYP3A5*3 and CYP3A4*22 alleles, the *CYP3A* locus contains many more rare variants which are expected to play a role in enzyme activity and drug response. Using a repeated drug administration method Ozdemir et al. compared standard deviations for inter- and intra-person variation in the disposition of 10 different CYP3A4 substrates, including midazolam, and cyclosporine. Analyses of the disposition parameters of these orally administered substrates suggested that at least 60% of the variability in composite CYP3A4 activity is under genetic control (Ozdemir et al., 2000). Furthermore, substantial missing heritability in CYP3A pharmacogenomics has been reported (Klein and Zanger, 2013). A twin study compared the differences of metabolic similarity between monozygotic (MZ) and dizygotic (DZ) twins (Rahmioglu et al., 2011). In this cohort of 367 healthy twins, 66% (confidence interval: 50%–77%) of the induced CYP3A4 activity variation was found to be hereditary, while only ∼20% was explained by current clinical PGx based on a GWAS study (Oetting et al., 2018). In this review, we outline the current pharmacogenetics knowledge of CYP3A activity and discuss potential future directions to improve our genetic knowledge and ability to explain CYP3A variability.

### Known *CYP3A4* and *CYP3A5* Haplotypes

There are 34 defined *-haplotypes for the *CYP3A4* and five in the *CYP3A5* gene designated by PharmVar (https://www.pharmvar.org/). Recently, three *CYP3A5* alleles (CYP3A5*2, *4, and *5) have been reclassified as part of the CYP3A5*3 suballeles (Rodriguez-Antona et al., 2022). Among those haplotypes, the most widely studied genetic variant is the CYP3A5*3 allele (rs776746), characterized by a splice defect in intron 3. This loss-of-function variant generates a premature stop codon that causes a lower amount of functional protein (Kuehl et al., 2001). Notably, CYP3A5*3 allele is common but the allele frequency differs between ethnicities, with the frequency of approximately up to 0.92 in European Americans (EA) and fluctuating between 0.24 and 0.84 in non-European populations according to the PharmGKB frequency table (https://www.pharmgkb.org/page/cyp3a5RefMaterials). A CYP3A5*3/*3 diplotype results in a 10–30 fold lower CYP3A5 expression level compared to CYP3A5*1/*3 (Hustert et al., 2001a; Lin et al., 2002). In clinical practice, individuals are categorized into three distinct CYP3A5 activity groups: CYP3A5 expressors (carrying two *1 alleles), heterozygous expressors (carrying one *1 allele and one *3 allele) and non-expressors (carrying two *3 alleles) of which the last group is the most common. These predicted metabolizer phenotypes can help explain differences in drug metabolism. For example, the dose corrected tacrolimus concentration after transplantation in patients who carried at least one CYP3A5*1 allele was significantly lower compared to CYP3A5 non-expressor after the first month following transplantation (*1.49 ± 0.88* vs. 3.11 ± 4.27*, p =* 0.01), which lasted for the first year post transplantation (Zheng et al., 2004). For this reason, the CYP3A5*3 allele is included in the CPIC, DPWG, RNPGx (French National Network of Pharmacogenetics), and IATDMCT (International Association of Therapeutic Drug Monitoring and Clinical Toxicity) guidelines as being associated with tacrolimus metabolism leading to the recommendation to increase 1.5—2.5 fold initial dosage in *1 carriers (Swen et al., 2011; Birdwell et al., 2015; Picard et al., 2017). In addition, two other key haplotypes CYP3A5*6 (rs10264272, c.624G > A) and CYP3A5*7 (rs41303343, c.1035dup) are included in the guidelines (Birdwell et al., 2015). CYP3A5*6 causes a splicing defect, and CYP3A5*7 results in a frameshift. These two alleles have a frequency of 11%–19% in the African population but have not been observed or with extremely low frequency in non-African populations (https://www.pharmgkb.org/page/cyp3a5RefMaterials). Both haplotypes are associated with no CYP3A5 catalytic activity and contribute to tacrolimus pharmacokinetics variability (Campagne et al., 2018), explaining about 6% of the variability in tacrolimus trough concentrations in African American patients (Oetting et al., 2016).

Besides CYP3A5*3, CYP3A4*22, located in the most predominant isoform of *CYP3A*, is the second *-allele that is often used in clinical practice. The CYP3A4*22 allele (rs35599367) is characterized by a G > A substitution in intron 6, resulting in an increased formation of a truncated alternative splice variant *in vitro* (Wang and Sadee, 2016). The allele frequency in the Caucasian population ranges from 3% to −5%, which is higher than compared to other populations, for example less than 1% in the Asian population. Notably, patients carrying CYP3A4*22 had a 1.7 to five fold decreased CYP3A4 mRNA or protein expression level (Elens et al., 2011; Wang et al., 2011; Wang and Sadee, 2016), explaining 12% of CYP3A4 activity variability (Wang et al., 2011). Moreover, the reduced activity caused by the CYP3A4*22 allele was verified with several CYP3A substrates *in vivo* (Elens et al., 2012; Elens et al., 2013a; de Jonge et al., 2015), and its contribution to variability in CYP3A activity and its potential clinical usage were summarized by Elens et al. and Mulder et al. (Elens et al., 2013b; Mulder et al., 2021). While CYP3A4 is involved in the metabolism of many drugs, there are currently no clinical guidelines available that include *CYP3A4* genetic variants. This is potentially explained by the low frequency of the variants in combination with a relatively modest effect on enzyme function which makes it more difficult to ascertain a connection between genotypes and drug metabolism and clinical outcomes.

In addition to these two well-recognized haplotypes, there are 37 additional *-allele haplotypes defined for *CYP3A4* and *CYP3A5*. A recently published review presented a comprehensive summarization of all *CYP3A5* *-alleles (Rodriguez-Antona et al., 2022). Here we give an overview of all *CYP3A4* *-alleles. Notably, for the *CYP3A4*, most of *-alleles are characterized by core variants which are rare (Minor allele frequency (MAF) < 1%), or very rare (MAF <0.1%). The only exception is CYP3A4*36, for which the global frequency is up to 0.42 based on the 1,000 Genomes. All *CYP3A4* alleles and *in vivo* evidence of their potential impact on enzyme activity are shown in Table 1. As the impact of these alleles are not assigned in PharmVar, the summarized impact of the alleles is based on available literature with *in vivo* evidence. CYP3A4*8 was detected with whole-exome sequencing in a patient with a severe paclitaxel-induced peripheral neuropathy due to diminished CYP3A4 enzyme activity (Apellániz-Ruiz et al., 2015b). Notably, the CYP3A4*18 haplotype is associated with a decreased midazolam metabolism but also results in a gain-of-function in the clearance of certain substrates such as sex steroids. These substrate specific effects may be attributed to structural changes in substrate recognition sites that results in catalytic activity variation (Kang et al., 2009). Besides, CYP3A4*1G, which has been redesignated as CYP3A4*36, has been reported a substate-dependent impact on CYP3A4 activity as well (Yuan et al., 2011; Dong et al., 2012; He et al., 2014). Notably, CYP3A4*1G has high linkage disequilibrium with CYP3A5*3 and significantly related with a lncRNA, AC069294.1, that caused down-regulated CYP3A4 and CYP3A5 expression (Collins and Wang, 2022). Furthermore, the interaction of CYP3A4*1G and CYP3A5*3 on drug pharmacokinetics, for instance, tacrolimus (Miura et al., 2011; Zuo et al., 2013) and sirolimus (Zhang et al., 2017) has been reported. For CYP3A4*20, a loss-of -function allele caused by a frameshift variant, it has been suggested that this variant causes an equal functional alteration as CYP3A4*22, given that in CYP3A5 non-expressors the phenotype of a heterozygous CYP3A4*20 carrier was close to that of a CYP3A poor metabolizer (Lloberas et al., 2018). Moreover, CYP3A4*20 presents a higher frequency and founder effect in the Spanish population, which highlights the contribution of rare *CYP3A* functional alleles in a specific population (Apellániz-Ruiz et al., 2015a). However, given the extremely low frequency of most of those additional *-alleles, there is not enough evidence for their functionality *in vivo* and for the clinical impact of these *-alleles. Several studies assess activities of these alleles *in vitro* (Fang et al., 2017; Xu et al., 2018; Li et al., 2019; Lin et al., 2019; Yang et al., 2019; Kumondai et al., 2021). Notably, their predicted function is shown to differ between substrates. For instance, the intrinsic clearance values of CYP3A4*14 and CYP3A4*15 haplotypes were higher compared to wild type for regorafenib (Li et al., 2019) and cabozantinib (Lin et al., 2019). By contrast, intrinsic clearance values were decreased for ibrutinib (Xu et al., 2018). Moreover, Ketoconazole, a CYP3A4 inhibitor alters the function of CYP3A4*14 and CYP3A4*15 in vitro (Lin et al., 2019), which warranted drug-drug interaction plays a significant role in observed enzyme activity. Besides, the variability of enzyme activity caused by some of those *-alleles might be too moderate to be identified, especially when influenced by diverse genetic and non-genetic factors *in vivo*. As a result, most of the currently reported haplotypes in *CYP3A4* and *CYP3A5* failed to be taken into account in clinical practice.

**TABLE 1:** Overview of core variants of *CYP3A4* haplotype^a^.

Allele	rs ID	Nucleotide changes (cDNA)	Amino acid changes	VEP annotation	*In vivo* evidence of function variability
*2	rs55785340	664T > C	S222P	Missense	
*3	rs4986910	1334T > C	M445T	Missense	
*4	rs55951658	352A > G	I118V	Missense	
*5	rs55901263	653C > G	P218R	Missense	
*6	rs4646438	830_831 insA	D277fs	Frameshift	
*7	rs56324128	167G > A	G56D	Missense	
*8	rs72552799	389G > A	R130Q	Missense	Decreased CYP3A4 activity Apellániz-Ruiz et al. (2015b)
*9	rs72552798	508G > A	V170I	Missense	
*10	rs4986908	520G > C	D174H	Missense	
*11	rs67784355	1088C > T	T163M	Missense	
*12	rs12721629	1117C > T	L373F	Missense	
*13	rs4986909	1247C > T	P416L	Missense	
*14	rs12721634	44T > C	L15P	Missense	
*15	rs4986907	485G > A	R162Q	Missense	
*16	rs12721627	554C > G	T185S	Missense	
*17	rs4987161	566T > C	F189S	Missense	
*18	rs28371759	878T > C	L293P	Missense	Increased tacrolimus clearance Liu et al. (2017), increased cyclosporine clearance Xin et al. (2014)
*19	rs4986913	1399C > T	P467S	Missense	
*20	rs67666821	1461_1462 insA	P488fs	Frameshift	Decreased tacrolimus clearance Gómez-Bravo et al. (2018)
*21	rs201821708	956A > G	Y319C	Missense	
*22	rs35599367	522-191C > T		Intron variant	Decreased enzyme activity^b^
*23	rs57409622	484C > T	R162W	Missense	
*24	rs113667357	600A > T	Q200H	Missense	
*26	rs138105638	802C > T	R268X	Missense	Decreased tacrolimus clearance Werk et al. (2014)
*28	rs570051168	64C > G	L22V	Missense	
*29	rs1449865051	337T > A	F113I	Missense	
*30	rs778013004	388C > T	R130X	Missense	
*31	rs1303250043	972C > A	H324Q	Missense	
*32	rs368296206	1004T > C	I335T	Missense	
*33	rs756833413	1108G > T	A370S	Missense	
*34	rs774109750	1279A > G	I427V	Missense	
*35	rs188389063	7C > G	L3V	Missense	
*36	rs2242480	1026 + 12G > A		Intron variant	Substrate-dependent^b^
*37	rs35599367 rs4986910	522-191C > T, 1334T > C	M445T	Intron variant, missense	

a Allele definitions are based on PharmVar. *In vivo* functionality is based on literature as activity is not reported in PharmVar. Therefore, we performed a literature search for each CYP3A4 *-allele in the table with Pubmed.

b Explanations in detail were described in the tex.

### Rare Variants in the *CYP3A* Locus

As mentioned, genetic variants in the *CYP3A* genes are related to CYP3A enzyme activity and, thereby drug response. However, currently used PGx approaches focusing on the well-known *-alleles, explain only a small proportion of the overall variability in the pharmacokinetics of CYP3A substrates. A recently published twin study revealed that up to 73% of the variability in CYP3A activity is attributed to genetic factors but only a part of this could be explained by PGx (Matthaei et al., 2020). This missing heritability suggests a role of more impactful variants inside and outside the *CYP3A* locus beyond the currently known and used haplotypes. For instance, a study showed that rare variants in *CYP3A4* may account for up to 99% of the functional variability (Kozyra et al., 2017). This proportion is much higher than some other pharmacogenes, in which rare variants contribute to 30%–40% of the variability.

**FIGURE 1:**
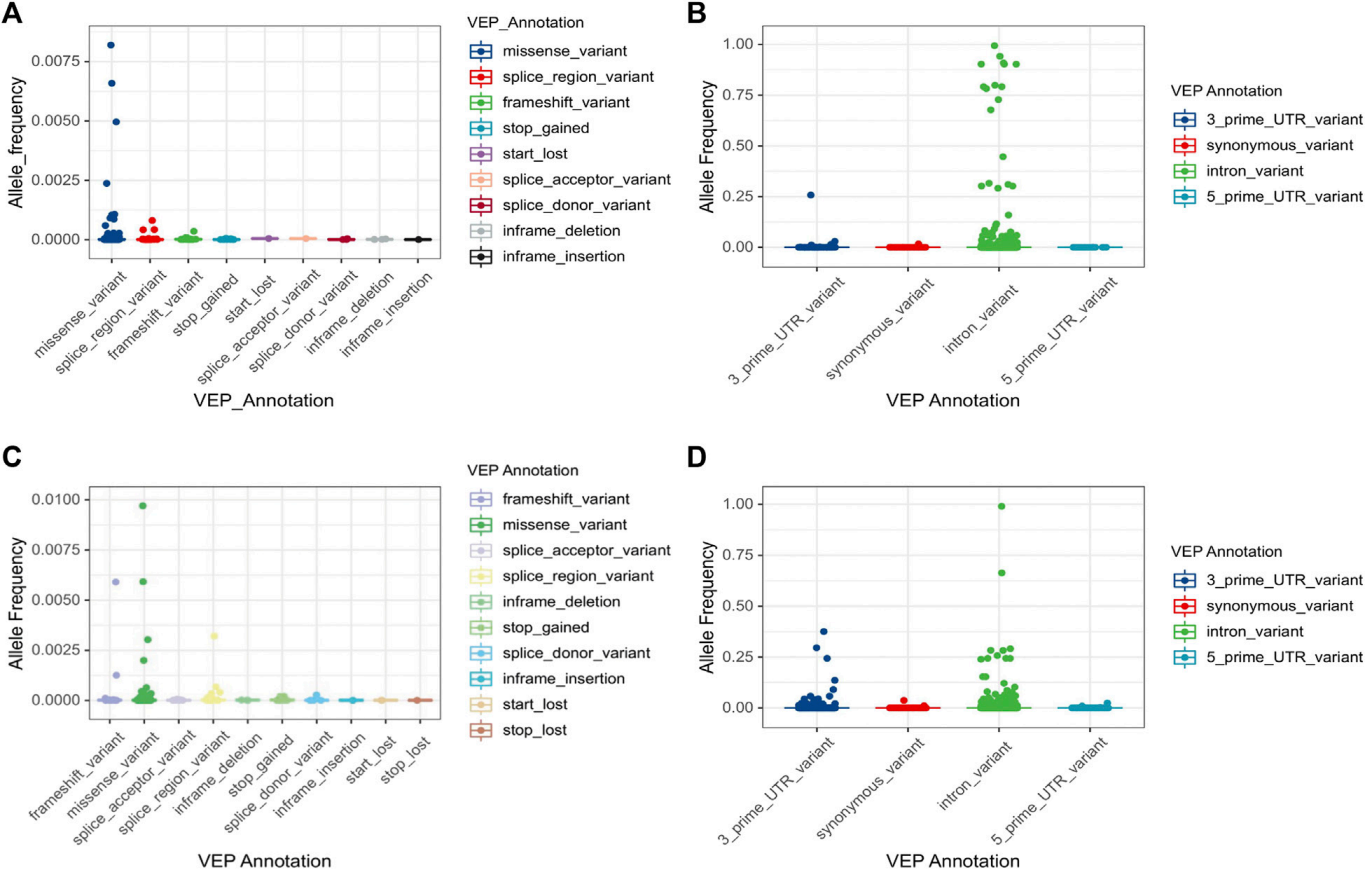
Allele frequency of *CYP3A4* and *CYP3A5* variations by VEP annotation. Total 5,082 variants in *CYP3A4* **(A,B)** and 6,035 variants in *CYP3A5* **(C,D)** are classified by VEP annotation. VEP annotations are separated by the impact on the coding sequence. Four common variants (MAF>1%) in *CYP3A5* are marked separately in Supplementary Figure S1. Note that stop lost variants were only reported in *CYP3A5*. VEP: Variant effect predictor.

Numerous genetic variants have been detected in the *CYP3A* locus, as shown in the data from The Genome Aggregation Database (gnomAD) (https://gnomad.broadinstitute.org/). More than 90% of genetic variants in this locus are rare, with a MAF of less than 1% (Table 2). In *CYP3A4*, a total of 5,082 variants are identified. Of these, only 85 are common (MAF >1%) (Figures 1A,B). All common variants are either located in non-coding regions or are synonymous variants, which do not result in amino acid changes. By contrast, all 285 non-synonymous variants are rare (MAF <1%) of these 218 are missense variants. In addition, there are 32 and 20 splice region variants and frameshift variants, respectively, which is more than the rest of the Variant Effect Predictor (VEP) annotation groups. Finally, there are over four thousand rare intronic variants. For *CYP3A5* (Figures 1A,C,D), a total of 6,151 variants are identified, of which 116 are common. Those common variants were mostly intronic or in the 3′untranslated region (3′-UTR) except for four variants which were non-synonymous (Figure 1D; Supplemental Figure S1). Moreover, 199 missense variants were detected in *CYP3A5*, all of which are rare, which is much more than other variant types. Notably, over 51% of all rare variants in the *CPY3A4* and *CYP3A5* genes were detected only in non-European populations. On the other hand, around 31% of all rare variants are limited to the European (non-Finnish) population [1576 (31.5%) and 1873 (31.0%) for *CYP3A4* and *CYP3A5* respectively]. These findings highlight the differences between ethnicities (https://gnomad.broadinstitute.org/).

**TABLE 2:** Summary of *CYP3A4* and *CYP3A5* allele number classified by VEP (variant effect predictor) annotation.

VEP Annotation	Number of variants in CYP3A4 N (%)			Number variants of in *CYP3A5* N (%)		
	All alleles	MAF<1%	MAF<0.1%	All alleles	MAF<1%	MAF<0.1%
Frameshift variant	20 (0.0057%)	20 (0.143%)	20 (0.474%)	24 (0.3997%)	23 (1.149%)	21 (0.183%)
Intron variant	4,472 (97.2661%)	4,393 (89.829%)	4,288 (87.122%)	4,641 (69.1792%)	4,559 (70.517%)	4,445 (77.117%)
Missense variant	218 (0.2206%)	218 (5.531%)	212 (4.541%)	199 (0.2909%)	199 (4.056%)	195 (2.935%)
Splice acceptor variant	1 (0.0004%)	1 (0.009%)	1 (0.030%)	8 (7.9761%)	7 (0.021%)	7 (0.069%)
Splice donor variant	3 (0.0004%)	3 (0.010%)	3 (0.034%)	5 (0.0032%)	5 (0.045%)	5 (0.147%)
Splice region variant	32 (0.0138%)	32 (0.346%)	32 (1.145%)	45 (0.1997%)	44 (0.872%)	43 (1.245%)
Start lost	1 (0.0004%)\	1 (0.009%)	1 (0.030%)	2 (0.0001%)	2 (0.002%)	2 (0.007%)
Stop gained	10 (0.0012%)	10 (0.030%)	10 (0.101%)	15 (0.0078%)	15 (0.108%)	15 (0.353%)
Stop lost	0	0	0	2 (0.7525%)	1 (0.001%)	1 (0.003%)
3′prime UTR variant	216 (2.3511%)	211 (3.402%)	205 (4.243%)	1027 (20.2284%)	1000 (20.770%)	968 (15.899%)
5′prime UTR variant	29 (0.0124%)	29 (0.310%)\	29 (1.025%)	108 (0.4166%)	107 (2.147%)	105 (1.033%)
Inframe deletion	4 (0.0004%)	4 (0.011%)	4 (0.037%)	3 (0.0004%)	3 (0.006%)	3 (0.020%)
Inframe insertion	1 (0.00004%)	1 (0.001%)	1 (0.004%)	1 (0.00007%)	1 (0.001%)	1 (0.003%)
Synonymous variant	75 (0.1275%)	74 (0.368%)	74 (1.215%)	71 (0.5452%)	69 (0.303%)	69 (0.987%)
Total	5,082 (100%)	4,997 (100%)	4,880 (100%)	6,151 (100%)	6,035 (100%)	5,880 (100%)

For both *CYP3A4* and *CYP3A5*, intronic variants are the most common type of variants, accounting for 97% and 69% of the single nucleotide variants (SNVs), respectively (Table 2). Although an intron is not translated, intronic variants are still of importance as they can influence splicing. For example, the core variant of the CYP3A4*22 allele is a deleterious intronic variant. Furthermore, rs4646450, located in the *CYP3A5* intron region, was associated with decreased protein expression and CYP3A4 activity, explaining 3%–5% of the variability (Klein et al., 2012). By contrast, most of common variants do not seem to cause alterations which can influence CYP3A activity. Moreover, a genome-wide associated study failed to identify any common variants related to induced CYP3A4 activity within 310 twins (Rahmioglu et al., 2013). Therefore, in the *CYP3A* locus, rare variants are the most likely to contribute profoundly to the variability of CYP3A enzyme activity. Notably, the log-transformed distribution of midazolam clearance showed a unimodal pattern (Lin et al., 2001), which suggests that the genetic impact on CYP3A activity could be attributed to a large number of small impact variants rather than only a few high impact ones.

### Genetic Factors Outside the CYP3A Locus

While variants within the genes in the *CYP3A* locus can influence CYP3A enzyme activity, variability in expression can also play a role. Transcriptional regulation of CYP3A expression has been reported in many studies (Yuan et al., 2020). Among those transcriptional factors, two predominant nuclear receptors, pregnane X receptor (*PXR, NR1I2*) and constitutive androstane receptor (*CAR, NR1I3*), have been repeatedly associated with altered CYP3A activity (Lolodi et al., 2017). These are ligand-activated nuclear receptors which, after being activated by specific ligands, heterodimerize with the retinoic acid receptor (*RXR; NR2B1*). Subsequently, they bind to the 5′ regulation region of target genes at the hormone-responsive elements (HREs) motif (Chen et al., 2012; Banerjee and Chen, 2013; Tebbens et al., 2018). Several HREs share the typical nuclear structure, implying the overlapping of downstream genes. But the expression regulation effect varies between target genes (Timsit and Negishi, 2007). Studies have shown that *CYP3A* isoforms are mainly regulated by *PXR* (Mbatchi et al., 2018). More functional nuclear receptor SNVs related to CYP3A activity and CYP3A substrate metabolism are summarized in Table 3. Moreover, the interaction between nuclear receptors also plays a role in CYP3A activity variation. Hepatocyte nuclear factor-4α (HNF4α) is a liver-enriched nuclear receptor and is associated with CYP3A4 and CYP3A5 expression (Jover et al., 2001). *HNF4α* not only regulates *CYP3A4* transcription directly by binding to two *CYP3A4* 5′ upstream regions featured with direct repeat (DR) 1-type motifs (Tegude et al., 2007) but also serves as a coactivator that interacts with the other two CYP3A4 regulators *PXR* and *CAR*, resulting in CYP3A4 expression variability. One of the *HNF-4α* polymorphisms (rs2071197), combined with PXR*1B, was related to the concentration-to-dose (C/D) ratios of carbamazepine, wild type *HNF-4α* carriers had higher C/D ratios in PXR*1B genotype rather than PXR*1B non-carriers (Saruwatari et al., 2014). Furthermore, the *CYP3A4* mRNA expression in pediatric livers could be better predicted with the model, including the expression level of *HNF-4α*, *PXR*, *CAR*, and their heterodimer partner *RXRα* (Vyhlidal et al., 2006). The interaction of transcriptional factors implies that the expression of CYP3A is the combined effect of multiple transcription factors. More *CYP3A* transcription regulators were reported recently. For instance, the *TSPYL* family suppressed the CYP3A4 expression and one SNV in *TSPYL1*, rs3828743, reversed the suppression effect (Qin et al., 2018). In addition to transcriptional regulation, the CYPP450 oxidoreductase (*POR*) is reported to influence CYP3A activity by participating in electron transfer to the CYP3A enzyme (Masters, 2005). Indeed several studies showed that a common variant POR*28 (rs1057868) was associated with lower C/D ratios and higher dose requirement of tacrolimus (de Jonge et al., 2011; Suetsugu et al., 2019; Nakamura et al., 2020) and cyclosporin A (Cvetković et al., 2017). In addition, epigenetic regulation of the CYP3A enzymes is an emerging research field (Tang and Chen, 2015). Several mechanisms have been revealed particularly in microRNA (miRNA) involved in transcriptional and post-transcriptional regulation (Wei et al., 2014; Ekström et al., 2015). Notably, miRNA not only targets the 3′-untranslated region (3′UTR) of CYP3A4 directly (Pan et al., 2009) but also impacts the function of CYP3A4 transcriptional factors, for instance, HNF4α (Takagi et al., 2010) and VDR (Pan et al., 2009). More mechanisms of epigenetic regulation and related miRNA have been reviewed but a detailed description is outside the scope of this manuscript (Dluzen and Lazarus, 2015; Lolodi et al., 2017). Overall, while some genetic factors outside the CYP3A locus have been linked to the variability of CYP3A4 expression or CYP3A activity, conflicting data are present and for none of the variants there is sufficient evidence to support clinical application.

**TABLE 3:** Summary of single nucleotide variants in CYP3A4 transcriptional factors associated with variability of CYP3A4 activity.

Gene	rsID	MAF (%)	Location	Nucleotide changes	Effect			Sample size	References
					Transcriptional regulator	*CYP3A* activity	Substrate metabolism		
*NR1I2 (PXR)*	rs3814058	21.1	3′UTR	T > C	Decreased expression	decreased	Decreased repaglinide clearance	300	Du et al., (2013)
	rs2276706	38.3	Intron1	G > A	Decreased expression	decreased	No effect on everolimus metabolis*	300 53*	Moes et al. (2012); Du et al. (2013)
	rs1523127	58.9	5′UTR	C > A	No change in RNA expression	Increased *CYP3A4* mRNA expression*	No effect on tacrolimus clearance	52* 336	King et al. (2007); Wang et al. (2016)

	rs3814055	38.3	5′UTR	C > T	Higher PXR promotor activity (*in vitro)*		No effect or decreased tacrolimus clearance*	35 32* 240*	Lamba et al. (2008); Benkali et al. (2009); López-Montenegro Soria et al. (2010); Kurzawski et al. (2017); Rana et al. (2017)
	rs2472677	44.9	Intron 1	C > T	Higher mRNA level	Increased*	Increased Atazanavir apparent clearance^#^	16 45* 109^#^	Hustert et al. (2001b); Lamba et al. (2008); Siccardi et al. (2008)
	rs1523130	58.2	5′UTR	T > C		Decreased		128	Lamba et al. (2008); Lamba et al. (2010)
	rs13085558	14.1	Intron 1	T > C		Increased (Female)		17	Lamba et al. (2008)
	rs3732359	73.8	3′UTR	G > A		Increased	Increased midazolam clearance	53	Oleson et al. (2010)
	rs3732360	71.3	3′UTR	C > T/C > G		Increased	Increased midazolam clearance	53	Oleson et al. (2010)
	rs3814057	19.6	3′UTR	A > C/A > T	Increased *PXR* mRNA expression*		Affected the concentration of Voriconazole	44* 172	Oleson et al. (2010); Zeng et al. (2020)
	rs7643645	34.9	Intron 1	A > G	Decreased *PXR* mRNA expression	Decreased *CYP3A4* mRNA level		16	Hustert et al. (2001b); Lamba et al. (2008)
	rs6785049	58.3	Intron 5	G > A		2-fold higher *CYP3A4* expression	Decreased Tacrolimus clearance	83	Stifft et al. (2018)
*NR1I3 (CAR)*	rs11265572	1.0	5′flanking region	G > T			Increased log-transformed tacrolimus C/D ratios	96	Chen et al. (2014)
	rs201406656	0.006	Exon7	A > G	Lower *CYP3A4* transactivation effect (*in vitro*)				Ikeda et al. (2005)
	1–161229921-A-G^a^	0.0007	Exon9	A > G	Lower *CYP3A4* transactivation effect (*in vitro*)				Ikeda et al. (2005)
*NR1I1 (VDR)*	rs1544410	38.8	Intron9	G > A/G > T/G > C		Decreased intestine *CYP3A4* expression		30	Thirumaran et al. (2012)
	rs4516035	39.9	Intron1	A > G		Higher *CYP3A4* expression and activity		210	Thirumaran et al. (2012); Nylén et al. (2014)
	rs11568820	28.1	Intron1	G > A	Decreased transactivation of *VDR* promoter	Higher *CYP3A4* expression		30	Thirumaran et al. (2012)
	rs731236	38.7	Exon 9	A > G	Decreased 60% *VDR* gene expression	No association with mRNA expression of *CYP3A4*		114	Betts et al. (2015)
*HNF4a*	rs2071197	10.8	Intron 1	G > A	G allele may diminish *HNF-4α* expression		Associated with CBZ concentrations stratified by PXR*1B	168	Saruwatari et al. (2014)
*NR3C1 (GR)*	rs258747	51.4	3′flanking region	A > G		Lower CYP3A4 activity		148	Klein et al. (2012)
*PPARA*	rs4253728	25.1	Intron3	G > A	AA homozygotes had 1.6-fold lower PPARA protein level*	Explained 5% CYP3A4 activity variation	Lower 2-OH-atorvastatin/atorvastatin AUC_0–∞_ ratio *in vivo*	46* 148	Klein et al. (2012); de Keyser et al. (2013)
	rs4823613	28.2	Intron3	A > C/A > G		Explained 9% CYP3A4 activity variation		148	Klein et al. (2012)
*ARNT*	rs2134688	89.1	Intron4	G > A		Lower *CYP3A4* mRNA and protein expression		150	Klein et al. (2012)
*TSPYL1*	rs3828743	24.1	Exon1	G > A/G > T		Increased *CYP3A4* expression (*In vitro*)	Increased abiraterone clearance (*in vitro*) and decrease the response rate of abiraterone* (*in vivo*)	89*	Qin et al. (2018)
*PGRMC2*	rs3733260	18.8	Intron1	G > T		Decreased CYP3A4 activity		147	Klein et al. (2012)

a There is no rsID available for this variants in the database.

### Opportunities for Pharmacogenomics Studies on CYP3A Missing Heritability

**FIGURE 2:**
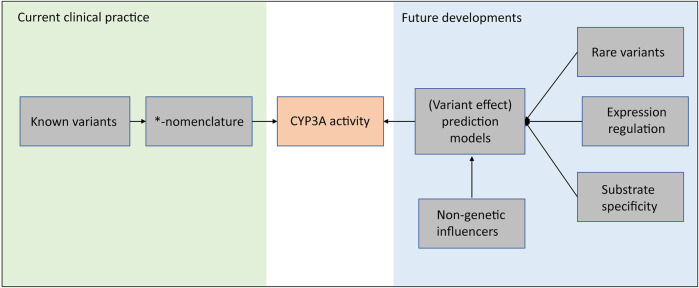
A Framework of future strategy for the CYP3A enzyme activity prediction.

Based on a genome-wide association study (GWAS) consisting of 1,446 kidney transplant recipients, 12.5% of tacrolimus trough concentration variability can be explained by CYP3A5*3 variants, including both donor factors and recipient factors. The explained variability increases to 16.9% after taking CYP3A4*22 into account (Oetting et al., 2018). This study combined with the two twin studies mentioned above encourages further exploration of the missing heritability in *CYP3A4*. And as discussed in this review, many of the variants in the CYP3A locus are of unknown impact and are not included in the clinical *-nomenclature. Moreover, not for all known *-haplotypes the impact is known. This limits the implementation of these variants and haplotypes in clinical practice. As shown in Figure 2, in our opinion, these future developments should focus on a unifying approach that incorporates all (genetic) factors which can influence CYP3A activity. For example, advanced prediction models, e.g., neural network models, which incorporate rare variants, expression regulation and non-genetic contributions, are expecting as a promising strategy for future developments.

However, currently studies in genetic variants detection and interpretation have inevitable limitations. GWAS analysis identified some genetic variants associated with the variability of CYP3A4 activity. However, by design GWAS analyses exclude rare variants and sometimes even low-frequency variants (MAF<5%) for reasons of quality control. As a result, GWAS is incapable to identify rare variants associated with clinical outcomes. Moreover, GWAS analysis generally relies on SNV screening array, which has difficulty detecting variants in complex genes and cannot detect novel variants. However, these types of variants are abundantly present in the *CYP3A* locus.

The complexity of the *CYP3A* locus encourages the application of advanced sequencing technologies. First of all, next-generation sequencing (NGS) provides us with almost all of the SNVs in the *CYP3A* locus. However, NGS generally relies on short reads (100–200 bp), which makes it limited in characterizing complex regions extending that length. Moreover, short-read sequencing still has limitations in exploring the structural variation and providing phasing information directly (Russell and Schwarz, 2020; van der Lee et al., 2020). Besides NGS, long-read sequencing is a promising solution for comprehensive genetic information collection (Ameur et al., 2019). With long-read sequencing, all single-nucleotide variants and most of the structural variants can be detected and those variants can often be phased directly, which is crucial for phenotype prediction (van der Lee et al., 2020). The accuracy and size of phased haploblocks of long-read sequencing seems most prominent in the complex pharmacogenes. This information can also be used to optimize models to predict drug response by taking phasing and full gene sequencing into account. Indeed, recently a continuous scale model based on full gene variants data explored by long-read sequencing and neural network improved the explanation of CYP2D6 activity variability from 54% to 79% compared with the conventional phenotype classification (van der Lee et al., 2021).

Nonetheless, these sequencing technologies come with higher costs and significantly more data to analyze, resulting in consideration of cost-benefit balance and the challenging of novel variant interpretation. To assess the individual impact of a large number of low frequency variants, and in the absence of *in vivo* data, several *in silico* tools, including SIFT, PolyPhen-2, and CADD have been developed. However, the predicted effect of variants differed among *in silico* tools (Gulilat et al., 2019). Moreover, compared with *in vitro* models, the accuracy of those *in silico* prediction only reaches up to 80% for pharmacogenetic purposes (Han et al., 2017). SIFT, PolyPhen-2 show a higher false-negative rate in predicting gain-of-function variants compared to loss-of-function (Flanagan et al., 2010). Remarkably, neural network models have been developed to predict the unknown function *-alleles in *CYP2D6*. This model explains 47.5% of unknown function variants in *-alleles with 88% accuracy (McInnes et al., 2020), which encouraged the further implementation of neural network based approaches. By contrast, *in vitro* models provide us a better understanding of the effects of variants, especially for rare variants (Kumondai et al., 2021). However, it is a costly and time-consuming process to establish *in vitro* models for each individual *CYP3A* variant, which can probably never be realized and hampers their clinical implementation. Furthermore, by-design an *in vitro* system is not useful to assess the effect of variants located in non-coding regions, which occur frequently in the *CYP3A* locus.

Given the broad substrate spectrum of drugs affected by the CYP3A enzymes and the presence of substrate-specific effects, functional prediction based on *in vitro* data with only a limited number of substrates are controversial. Therefore, *in vivo* evidence is considered the gold-standard to establish if variants have a significant effect on enzyme activity, especially for non-coding and synonymous SNVs. For novel missense SNVs, drug-related clinical information is highly recommended. However, particularly for the frequently occurring low-frequency non-synonymous variants in the CYP3A locus, this evidence is hard to generate. Indeed, quite a few studies failed to detect functional variants or identify their associations with clinical outcomes (Belmonte et al., 2018; Riera et al., 2018). Moreover, lacking of clinical significance prevent their further study only based on *in vitro* evidence.

Conventional drug metabolizer phenotypes distinguish three or four metabolizer phenotype groups based on a limited number of variants. This classification assumes a fixed effect of each variant, where the predicted phenotype depends on the combined effects of those several well-known variants. This same method is used for almost all CYP-enzymes with in the end only five phenotype categories (poor-, intermediate-, normal-, rapid- and ultra-rapid metabolizers) (Caudle et al., 2017). However, it has been demonstrated that enzyme activity is not categorical but continuous. As a result, the conventional variant to metabolizer phenotype interpretation which only includes a limited number of well-known variants with fixed effects inevitably leads to missing information. Previous studies have shown that, at least for CYP2D6, a continuous model is able to better explain the enzyme activity (McInnes et al., 2020; van der Lee et al., 2021). It can be expected that the same principle holds true for CYP3A as well. Furthermore, in the *CYP3A* locus more than one coding isoforms with considerable overlapped substrate specificity contributes to the overall CYP3Aenzyme activity, complicating the predictions even more. As mentioned previously, CYP3A mediated clearance shows a unimodal distribution which does not fit the current categorical phenotype system. In the current system, the function of variants that cause slight or moderate CYP3A activity variability could be obscured by variants that have predominant effects, which further complicates the analysis of the contribution of every variant in every *CYP3A* gene. To deal with the complexity of the *CYP3A* locus, the use of artificial intelligence (AI) is an opportunity. AI models could include all variants detected to predict both variant effect as well as overall enzyme activity without the use of the categorical models (Zou et al., 2019).

Besides genetic variation, environmental factors also contribute to variability in CYP3A activity. For instance, 20% of induced CYP3A4 activity variation was attributed to BMI, alcohol use, and smoking (Rahmioglu et al., 2011). Also, inflammation has been shown to affect CYP3A activity through modulation of the expression (Vet et al., 2016). Moreover, some clinical variables, including transplant recipient age, glomerular filtration rate, anti-cytomegalovirus drug use, simultaneous pancreas-kidney transplant and antibody induction, account for 19.8% of the variability in tacrolimus trough concentrations (Oetting et al., 2016). Finally, CYP3A inducers and inhibitors have been shown to cause a ∼400-fold fluctuation in CYP3A activity and drug clearance (Hohmann et al., 2016). Zanger et al. summarized these and other non-genetic factors which are beyond the scope of this review (Zanger and Schwab, 2013). In summary, genetic variability in the *CYP3A* locus has been consistently linked with the inter-individual variability of drug metabolism and differences in drug response. Despite indications for clinical relevance, only a limited number of variants are included in CYP3A metabolizer phenotype prediction and clinical practice. The abundance of rare variants in the *CYP3A* locus and possible multi-genic regulations of CYP3A expression combined with the rapid technological advances in sequencing technologies present an opportunity for future investigations and offer a potential explanation for the observed missing heritability.

## Supplementary Material

Supplementary Figure S1 The allele frequency of four common *CYP3A5* variants with potential impact VEP annotation.3.
