# Supplementary figures and images for: Why We Need to Take a Closer Look at Genetic Contributions to CYP3A Activity
Source: Front Pharmacol. 2022 Jun 16;13:912618. doi: 10.3389/fphar.2022.912618 (PMC9243486; doi:10.3389/fphar.2022.912618)

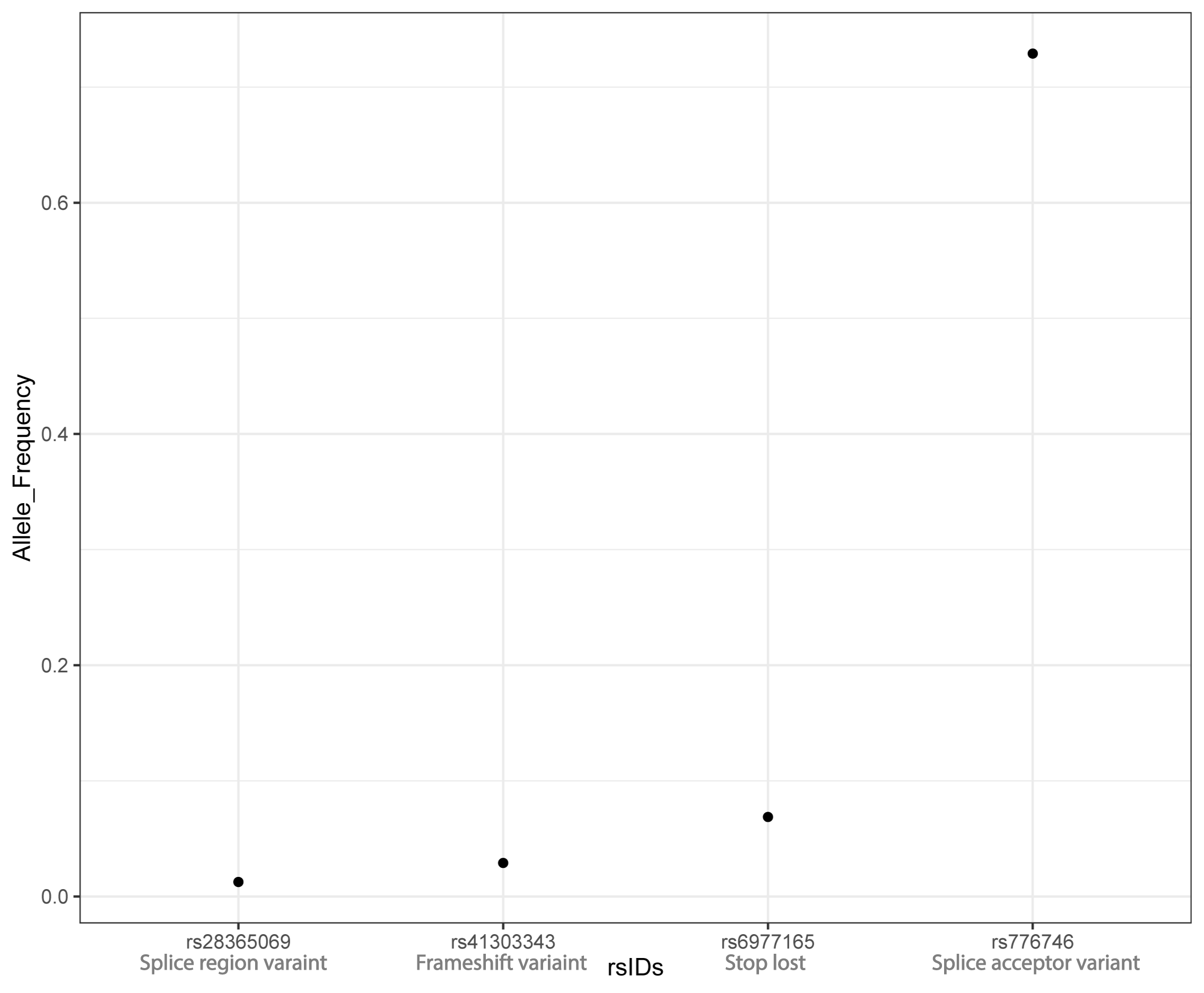

Supplement: Supplementary file 1 [file Image1.TIF]
